# Enhancing Early Detection of Aortic Coarctation With Perfusion Index: A Report of Two Cases

**DOI:** 10.7759/cureus.76866

**Published:** 2025-01-03

**Authors:** Patrícia Gomes Pereira, Cláudio Henriques, Filipa Vilacova, Sílvia Alvares, Elisa Proença

**Affiliations:** 1 Pediatrics, Unidade Local de Saúde da Região de Aveiro, Aveiro, PRT; 2 Department of Neonatology, Neonatal Intensive Care Unit, Centro Materno Infantil do Norte, Unidade Local de Saúde de Santo António, Porto, PRT; 3 Department of Pediatric Cardiology, Centro Materno Infantil do Norte, Unidade Local de Saúde de Santo António, Porto, PRT; 4 Unit for Multidisciplinary Research in Biomedicine, School of Medicine and Biomedical Sciences of Porto University, Porto, PRT

**Keywords:** aortic coarctation, congenital heart defects, critical congenital heart defects screening, left heart obstructive diseases, perfusion index

## Abstract

The perfusion index (PI) is a non-invasive method to assess peripheral perfusion, derived from and displayed by pulse oximeters. While pulse oximetry demonstrates moderate sensitivity in detecting obstructive left heart lesions, including coarctation of the aorta (CoA), its limitations underscore the need for improved screening techniques. This report presents the cases of two full-term neonates diagnosed with CoA, highlighting the role of PI in early detection before clinical signs such as pulse discrepancies and blood pressure gradients become apparent. Both neonates exhibited abnormal PI readings (low post-ductal PI and a significant pre- to post-ductal PI differential) prior to the emergence of traditional diagnostic signs of CoA. Both patients were treated with prostaglandin E1 and underwent corrective surgery. Although further research is required and PI is not yet recommended as a standard screening parameter for congenital heart defects, monitoring microcirculation through PI shows promise for earlier detection of left heart obstructive defects like CoA.

## Introduction

Congenital heart defects (CHDs) are a leading cause of morbidity and mortality in newborns. Among these, coarctation of the aorta (CoA) stands out as a critical condition, with a prevalence of approximately four per 10,000 live births [1]. It is one of the most significant left heart obstructive diseases, characterized by a narrowing of the aorta that restricts blood flow and increases cardiac workload. Early diagnosis of critical CHDs (CCHDs), including CoA, is essential for timely intervention, underscoring the importance of effective neonatal screening methods [1].

Traditionally, postnatal pulse oximetry screening (POS) and clinical examination have been the primary diagnostic tools for CoA. However, the condition often presents diagnostic challenges in the neonatal period due to its variable timing of presentation and subtle early signs. Key diagnostic clues include differences in pulses and blood pressure (BP), while confirmation typically relies on echocardiography. Despite this, traditional methods can lead to missed or delayed diagnoses, especially in cases of left heart obstructive defects like CoA. Although POS is a valuable tool, it does not reliably detect all newborns with CCHDs, particularly CoA [1-2].

This diagnostic gap has driven interest in developing reliable, objective, and non-invasive screening tools to improve detection rates. In this context, the perfusion index (PI) has emerged as a promising addition to neonatal screening. PI, derived from pulse oximetry, is a non-invasive real-time measure of peripheral blood flow that offers insights into circulatory status. There is a high degree of heterogeneity in PI values and reference values can be influenced by gender and birth weight [3]. By identifying reduced blood flow associated with left heart obstruction, PI demonstrates low false-positive rates and holds potential as a routine screening tool for CCHD. This is especially pertinent for CoA, where altered perfusion gradients can provide critical diagnostic clues [2,4].

Recent studies have highlighted PI's efficacy as a screening metric, particularly for detecting neonatal CoA. When combined with other screening modalities, PI improves early detection rates and minimizes delays in diagnosis and treatment. As a non-invasive and accessible indicator of critical left heart obstruction, PI may enhance neonatal outcomes [5].

## Case presentation

Case 1

The mother was a 36-year-old healthy primigravida. At 30 weeks and four days of gestation, a fetal ultrasound revealed right heart chamber predominance. Subsequent fetal echocardiography identified a "gothic" aortic arch with a narrow isthmus and turbulent flow. The pregnancy culminated in a vaginal delivery at 40 weeks and one day, with meconium-stained amniotic fluid. The male newborn, appropriate for gestational age, weighed 3250 g and had Apgar scores of 9, 10, and 10 at the first, fifth, and 10th minutes, respectively. Despite normal physical status, he was admitted to the neonatal intensive care unit (NICU) for surveillance due to prenatal findings.

**Figure 1:**
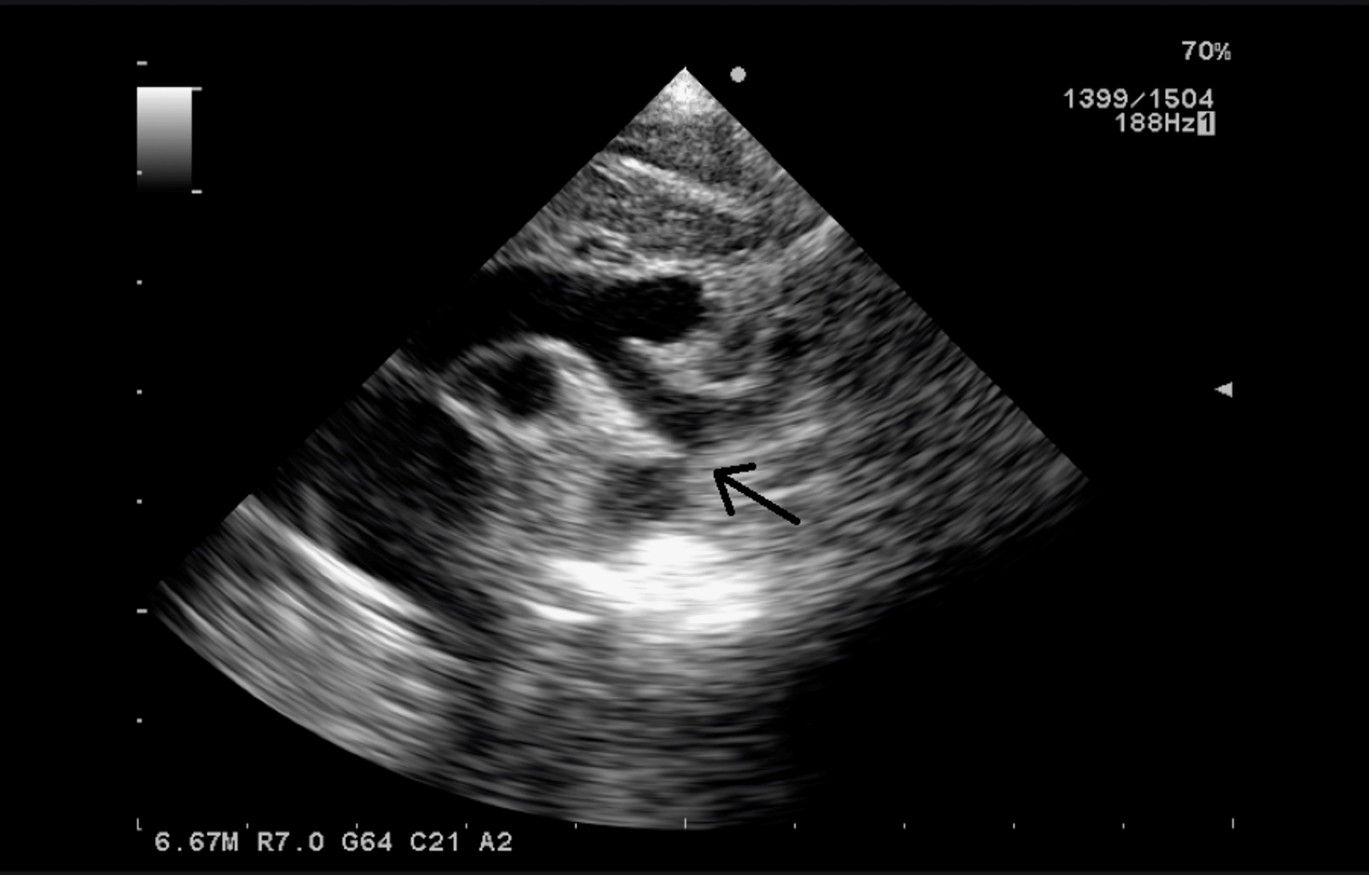
Echocardiography (Eco 2D/ Doppler) on day 1 of life (Case 1): a “gothic” aortic arch with isthmus narrowing (arrow).

On day 1, the neonate remained hemodynamically stable without cardiac murmurs or pulse abnormalities. Pulse oximetry readings showed pre- and post-ductal oxygen saturation (SpO₂) of 97-98%, with no BP differentials. Echocardiography confirmed right heart chamber and pulmonary artery predominance, a tortuous "gothic" aortic arch with isthmus narrowing and turbulent flow, a patent ductus arteriosus (PDA) with left-to-right shunt, and a patent foramen ovale (PFO) with minimal left-to-right shunt (Figure 1).

**Figure 2:**
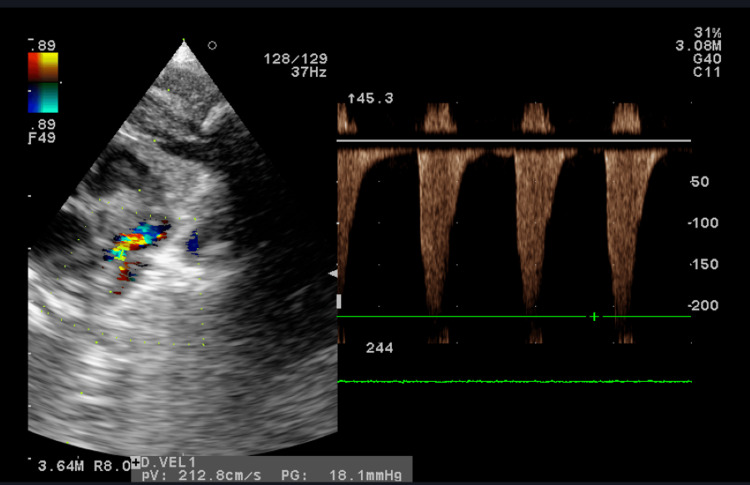
Echocardiography (Eco 2D/ Doppler) on day 3 (Case 1): turbulence distal to the subclavian artery with a gradient of 18 mmHg, without CoA flow, and a closed ductus arteriosus. CoA: coarctation of the aorta

By day 3, the neonate remained stable with no cardiac murmurs and normal femoral pulses. SpO₂ readings were 99% pre-ductal and 100% post-ductal, with no BP differential. However, PI readings showed pre-ductal values of 1.20, 1.77, and 1.58, respectively, compared to post-ductal values of 0.35, 0.53, and 0.36 (Table 1). Echocardiography revealed turbulence distal to the subclavian artery with a gradient of 18 mmHg, though no CoA flow was observed, and the ductus arteriosus had closed (Figure 2).

**Table 1:** PI, SpO₂, and blood pressure (Case 1) PI: perfusion index; SpO_2_: oxygen saturation

Day of Life	Pre-ductal PI	Post-ductal PI	Pre-ductal SpO₂ (%)	Post-ductal SpO₂ (%)	Blood Pressure
3	1.28- 1.77	0.35-0.53	99	100	Normal and no differential
5	2.0	0.54	99	99	Differential > 15 mmHg

**Figure 3:**
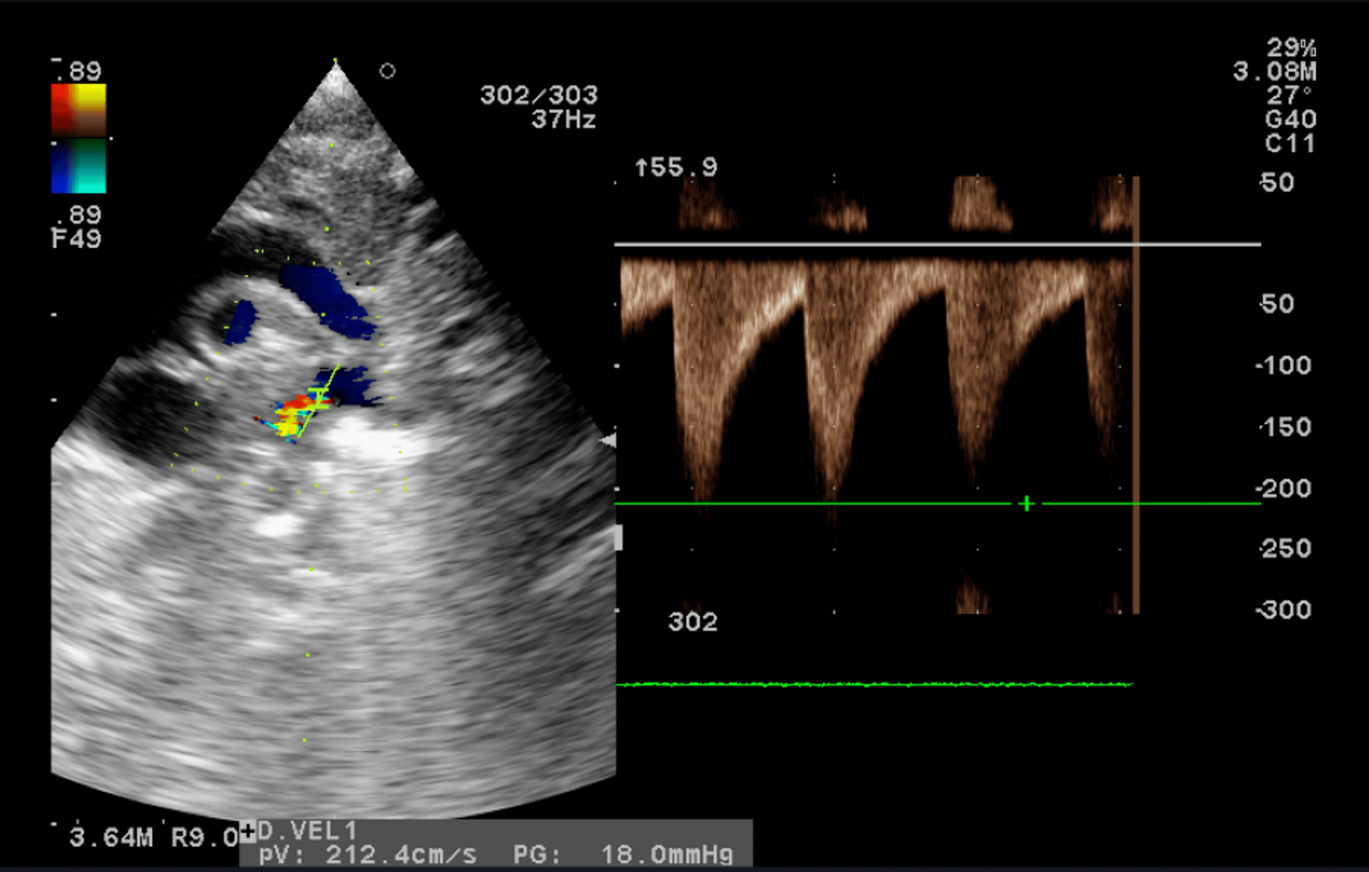
Echocardiography (Eco 2D/ Doppler) on day 5 of life (Case 1): CoA flow on aortic isthmus CoA: coarctation of the aorta

On day 5, despite stable SpO₂ readings (pre-ductal 98%, post-ductal 99%), a cardiac murmur was noted alongside weak femoral pulses and a BP differential of approximately 15 mmHg. PI readings demonstrated a marked pre- to post-ductal difference (2.0 vs. 0.54). Echocardiography confirmed the presence of CoA (Figure 3). The newborn underwent corrective surgery on day 6 and had an uneventful recovery.

Case 2

The mother, a healthy 41-year-old woman in her fourth pregnancy, underwent fetal echocardiography at 30 weeks of gestation, revealing subaortic and muscular ventricular septal defects (VSDs) and ductus venosus agenesis. The delivery was vaginal with vacuum assistance at 38 weeks and four days. The female newborn, appropriate for gestational age, weighed 2770 g and had Apgar scores of 8, 7, and 8 at the first, fifth, and 10th minutes, respectively. She required positive-end-expiratory pressure (PEEP) for approximately 20 minutes due to respiratory distress and was admitted to the NICU.

**Figure 4:**
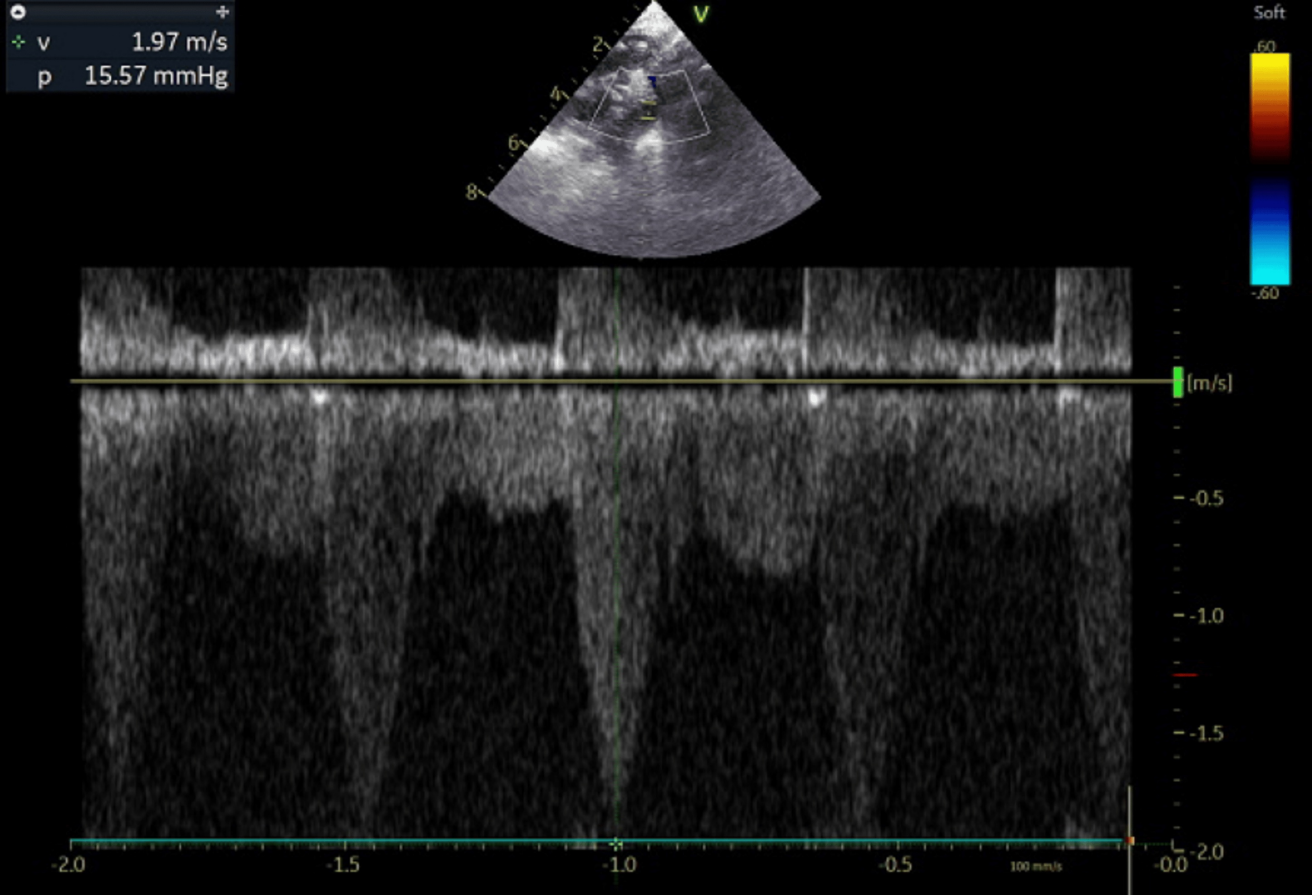
Echocardiography (Eco 2D/ Doppler) in the first hours of life (Case 2): "gothic" aortic arch with flow acceleration at the isthmus with a gradient of 15 mmHg.

On day 1, she remained on oxygen support and transitioned to nasal continuous positive airway pressure (CPAP). A systolic murmur was noted, but femoral pulses were normal, with pre- and post-ductal SpO₂ of 97% and 100%, respectively, and no BP differential. Echocardiography confirmed multiple VSDs and a "gothic" aortic arch with isthmus flow acceleration (Figure 4).

**Figure 5:**
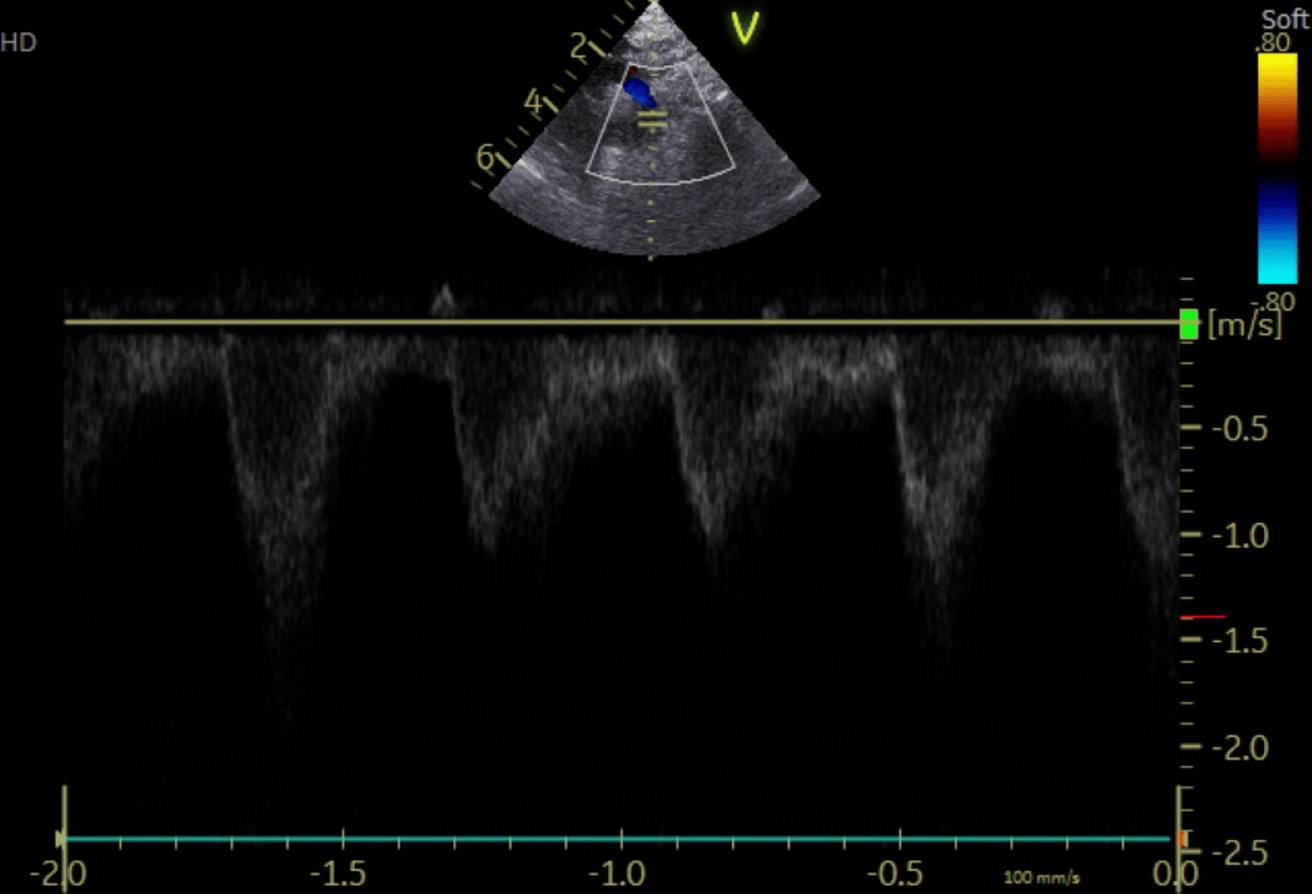
Echocardiography (2D/ Doppler) at approximately 36 hours of life revealed a patent ductus arteriosus with a left-to-right shunt and evidence of developing CoA flow (Case 2). CoA: coarctation of the aorta

By day 2, the infant required invasive ventilation due to increased oxygen needs. Femoral pulses remained normal, SpO₂ was stable, and BP measurements showed no differential. However, PI readings were 1.20 pre-ductal and 0.23 post-ductal (Table 2). Echocardiography at 36 hours revealed a small "gothic" aortic arch with slight hypoplasia of the transverse and descending aorta and developing CoA flow (Figure 5). Prostaglandin E1 infusion was initiated, and the diagnosis of CoA was confirmed. Despite undergoing corrective surgery on day 12, the newborn died from complications one month later.

**Table 2:** PI, SpO₂, and blood pressure (Case 2) PI: perfusion index; SpO_2_: oxygen saturation

Day of life	Pre-ductal PI	Post-ductal PI	Pre-ductal SpO₂ (%)	Post-ductal SpO₂ (%)	Blood Pressure
2	1.2	0.23	98	99	Normal and no differential

## Discussion

CoA is a CCHD that often presents diagnostic challenges in neonates, primarily due to its subtle and variable presentation [2]. Traditional diagnostic tools, such as pulse oximetry and clinical examination, can sometimes fail to detect CoA early enough for timely intervention. These cases highlight the complexity of early diagnosis and underscore the potential role of PI in identifying perfusion abnormalities before classic symptoms, such as weak femoral pulses or BP differentials, appear.

Both cases presented significant disparities between pre- and post-ductal PI values, which preceded the clinical manifestations of CoA. A low post-ductal PI (<0.7) and a pre- to post-ductal PI differential exceeding 50% were identified as early indicators of CoA, highlighting the potential of PI to detect circulatory changes associated with left heart obstruction. These findings are consistent with previous research suggesting that PI, when used in conjunction with other diagnostic modalities, can improve the early detection of CoA [1, 2].

Several studies have investigated the use of PI as a screening tool for neonatal CCHDs and revealed that PI demonstrated a low false-positive rate and was effective in screening for neonatal CoA, providing earlier detection than traditional pulse oximetry alone [1]. Additionally, noted that PI could be particularly useful in detecting left heart obstructions like CoA, where subtle changes in perfusion are critical for a diagnosis [5]. Although PI has not yet been universally adopted in neonatal screening, its potential as a supplementary tool is becoming increasingly recognized.

However, interpreting PI values in neonates requires careful consideration of various factors, including skin temperature, vasomotor tone, and potential motion artifacts, which can influence measurements [1]. Despite these challenges, the non-invasive nature of PI, combined with its ease of integration into existing neonatal screening protocols, makes it an attractive option for enhancing early diagnosis of CoA and other CCHDs.

The integration of PI into routine neonatal screening could provide several advantages. It is a simple and cost-effective method that can be performed as part of standard POS. Then PI values offer real-time insights into peripheral perfusion, which could help neonatologists make faster decisions about whether further diagnostic evaluation, such as echocardiography, is necessary. Although further research is required to standardize PI thresholds and determine its clinical utility across different populations, early indications are promising.

While echocardiography remains the gold standard for diagnosing CoA, the inclusion of PI in a multifaceted screening approach could improve early detection, thereby enabling timely intervention and potentially improving neonatal outcomes.

## Conclusions

The presented cases highlight the potential of the PI as a valuable tool for the early detection of CoA. By identifying perfusion abnormalities before the onset of classic clinical symptoms, PI offers an additional layer of diagnostic support, particularly for left heart obstructions where subtle changes in peripheral circulation are critical for early identification. However, widespread adoption requires further research to validate PI thresholds, address variability factors, and assess its clinical utility across diverse populations. Large-scale studies are needed to standardize its use and establish its role in comprehensive neonatal screening strategies.
